# Genipin Derivatives Protect RGC-5 from Sodium Nitroprusside-Induced Nitrosative Stress

**DOI:** 10.3390/ijms17010117

**Published:** 2016-01-19

**Authors:** Rikang Wang, Jiaqiang Zhao, Lei Zhang, Lizhi Peng, Xinyi Zhang, Wenhua Zheng, Heru Chen

**Affiliations:** 1 National Pharmaceutical Engineering Center for Solid Preparation in Chinese Herbal Medicine, Jiangxi University of Traditional Chinese Medicine, Nanchang 330006, China; wrk168ok@163.com; 2 Institute of Traditional Chinese Medicine and Natural Products, College of Pharmacy, Jinan University, Guangzhou 510632, China; jqzhaodzs@163.com (J.Z.); 13247377339@163.com (L.Z.); 15900088863@163.com (L.P.); zhangxinyi9205@163.com (X.Z.); 3 Faculty of Health Sciences, University of Macao, Macao, China; 4 Guangdong Province Key Laboratory of Pharmacodynamic Constituents of TCM and New Drugs Research, Guangzhou 510632, China

**Keywords:** NO neurotoxicity, neuroprotection, neurodegeneration, apoptosis, genipin, nitric oxide synthase

## Abstract

**CHR20** and **CHR21** are a pair of stable diastereoisomers derived from genipin. These stereoisomers are activators of neuronal nitric oxide synthase (nNOS) and endothelial nitric oxide synthase (eNOS). In the rat retinal ganglion (RGC-5) cell model these compounds are non-toxic. Treatment of RGC-5 with 750 μM of sodium nitroprusside (SNP) produces nitrosative stress. Both genipin derivatives, however, protect these cells against SNP-induced apoptic cell death, although **CHR21** is significantly more potent than **CHR20** in this regard. With Western blotting we showed that the observed neuroprotection is primarily due to the activation of protein kinase B (Akt)/eNOS and extracellular signal-regulated kinase (ERK1/2) signaling pathways. Therefore, LY294002 (a phosphatidylinositol 3-kinase (PI3K) inhibitor) or PD98059 (a MAPK-activating enzyme inhibitor) abrogated the protective effects of **CHR20** and **CHR21**. Altogether, our results show that in our experimental setup neuroprotection by the diasteromeric pair is mediated through the PI3K/Akt/eNOS and ERK1/2 signaling pathways. Further studies are needed to establish the potential of these compounds to prevent ntric oxide (NO)-induced toxicity commonly seen in many neurodegenerative diseases.

## 1. Introduction

Nitric oxide (NO), a Janus-faced molecule with pleiotropic effects in different tissues, is physiologically produced through the l-arginine/NO synthase (NOS) pathway. NOS is a family of four major types of enzymes including endothelial (eNOS), neuronal (nNOS), inducible (iNOS) and mitochondrial (mtNOSs). On the one hand, endogenous NO takes part in various physiological processes in the central nervous system, such as neuro-survival, neuro-differentiation, neuro-modulation, neuro-transmission and synaptic plasticity [1,2,3]. On the other hand, overproduction of NO produces oxidative and nitrosative stress [4,5,6,7]. Excess NO also induces nitrotyrosination, leading to the inhibition of tyrosine phosphorylation and blockade of the signal transduction pathways of growth factors [8]. Solid evidence supports that all neurodegenerative diseases are closely related to the overproduction of NO [9,10,11,12,13]. For example, when nNOS is activated with persistent stimulation of excitatory amino acid receptors mediating glutamate toxicity, as well as when iNOS is induced by diverse stimuli like endotoxin or cytokines, NO may probably be overproduced [9]. In these cases, the role of NO changes from a physiological neuromodulator to a neurotoxic factor.

No doubt, overproduction of NO induces oxidative stress. That is a risk factor for the development of neurodegenerative diseases including glaucomatous visual field deterioration [5,6,7]. Unfortunately, retinal ganglion cells (RGCs) are susceptible to oxidative stress that may trigger cell apoptosis [7,14,15]. In a variety of retinal degenerative diseases, such as glaucoma and diabetic retinopathy, this oxidative stress-induced RGC apoptosis is a fundamental pathogenesis [15,16,17]. Although the rat retinal ganglion (RGC-5) cell line is actually misidentified [18], we believe that it still possesses the fundamental properties of the retinal neuronal precursor cells and hence is suitable for our studies. In this way, RGC-5 cells can be used to study the pathogenesis process of oxidative/nitrosative stress induced by an exogenous nitric oxide agent, such as, sodium nitroprusside (SNP).

**Figure 1:**
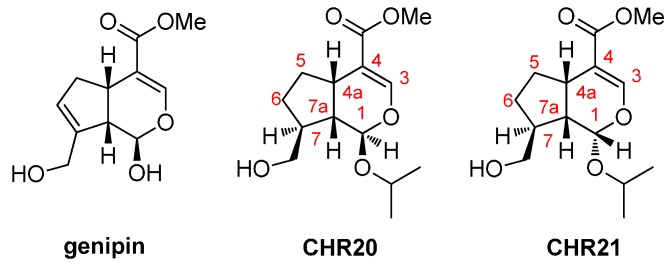
Chemical structures of genipin and **CHR20/21**. “a” is a symbol in the IUPAC’ nomemclature rule to represent the first atom linked to the outside ring.

Recently, application of several types of selective inhibitors of nNOS for the treatment of central nervous system (CNS) disorders have been reported [19,20,21,22]. Evidences based on the rodent model of dural inflammation relevant to migraine pain and the rat Chung model of neuropathic pain confirmed that some 3,4-dihydroquinolin-2(1*H*)-one and 1,2,3,4-tetrahydroquinoline-based derivatives had significant efficacy to relieve the pains [22]. As a matter of fact, our research group has synthesized and identified gardenamide A (GA), which was confirmed as an activator of nNOS and eNOS [23]. Quite interestingly, GA showed neuroprotective effects against serum deprived impairments in PC12 cells and H_2_O_2_-induced insults in RGC-5 cells [24,25]. The neuroprotective mechanisms involved are closely related to the activations of ERK1/2 and PI3K/Akt signaling pathways. Another two new stable genipin derivatives, **CHR20** (1-*R* isomer) and **CHR21** (1-*S* isomer), which are a pair of diastereoisomers, and confirmed as nNOS and eNOS activators have also been synthesized by us [26] (Figure 1). They protected PC12 cells from apoptosis induced by SNP. SNP was found to raise oxidative stress in PC12 cells and **CHR20/21** increase mRNA levels of glutamate-cysteine ligase catalytic subunit (GCLC) and superoxide dismutase 1 (SOD1) in a time-dependent manner [26].

In the present study we wished to investigate whether **CHR20** and **CHR21** can prevent RGC-5 cell death brought about by SNP-produced oxidative/nitrosative insults. 

## 2. Results and Discussion

### 2.1. Effects of SNP on RGC-5 Cells

**Figure 2:**
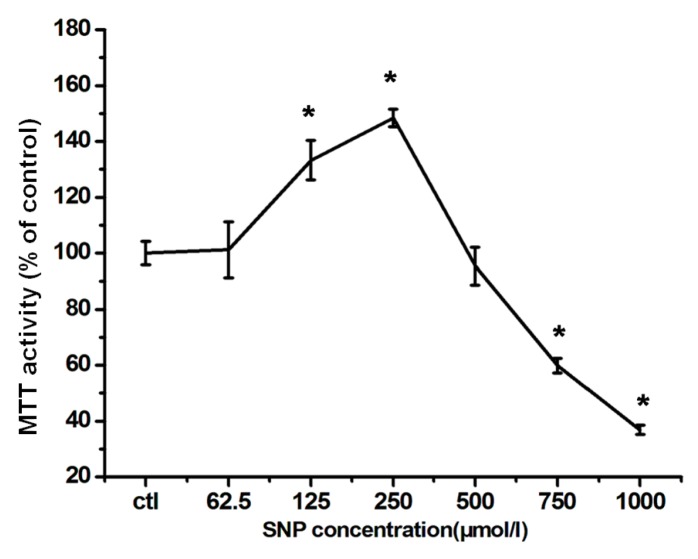
The neuro-effects of SNP on RGC-5 cells. Cells were exposed to different concentrations of SNP as indicated for 24 h. Cell viability was evaluated by MTT assay. The values were expressed as percentage of control, which is set to 100%. The percentage of MTT activity was presented as mean ± SD for six replicates. * *p* < 0.05 *vs.* control group.

As shown in Figure 2, SNP dose-dependently influenced RGC-5 viability. When RGC-5 cells were treated with SNP at a concentration between 62.5 to 250 μmol/L, their MTT activities increased with SNP concentration, in which MTT means 3-(4,5-Dimethylthiazol-2-yl)-2,5-diphenyl tetrazolium bromide. This increasing effect went to the maximal at 250 μmol/L. Because MTT activity is dependent on NADPH-dependent oxido-reductase enzymes largely in the cytosolic compartment of the cell [27,28], under defined conditions, it may reflect the number of present viable cells. Therefore, SNP at these concentrations showed no cytotoxicity to RGC-5 cells. Under these conditions, cell viability seems to increase with increasing SNP concentration. However, at high concentrations (750 and 1000 μmol/L), SNP was cytotoxic; exposure to 750 μmol/L of SNP induced injury to the cells. The MTT activity was only 41.0% ± 4.8% compared with that of the control group.

It is quite interesting that low concentration of SNP (≤250 µM) stimulates RGC-5 cell viability. We did observe cell proliferation under microscopy. However, the exact mechanism needs to be further investigated.

### 2.2. Cytotoxicity and MTT Activity Enhancement of **CHR20/21** to RGC-5 Cells

**Figure 3:**
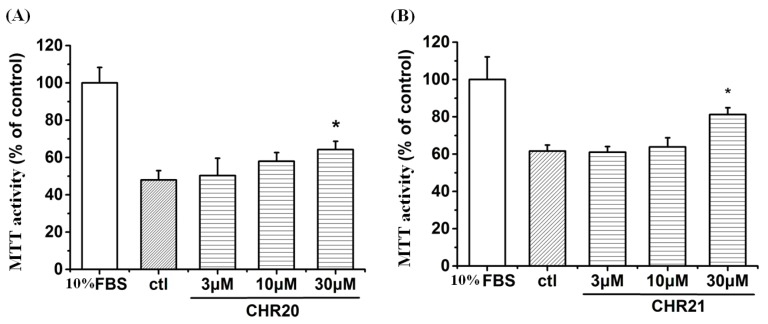
Cytotoxicity and MTT activity of **CHR20** and **CHR21** on RGC-5 cells. Cells that were cultured in RPMI-1640 medium with low serum (0.2% of FBS) for 24 h were set as the control group. RGC-5 cells with 0.2% FBS were treated with **CHR20** (**A**) and **CHR21** (**B**) at concentrations of 3, 10 and 30 µM, respectively for 24 h. Cell viability was determined by MTT assay and presented as mean ± SD for six replicates. * *p* < 0.05 *vs.* control group.

To examine the cellular tolerance to **CHR20/CHR21**, RGC-5 cells were cultured in RPMI-1640 medium with 10% fetal bovine serum (FBS) or low serum (0.2% of FBS) for 24 h. Then RGC-5 cells with low serum were treated with the test compounds at concentrations of 3, 10 and 30 μM, respectively, for 24 h. As shown in Figure 3, **CHR20** and **CHR21** did not show any toxicity at concentrations up to 30 μM. On the contrary, MTT activity of RGC-5 cells increased in the presence of **CHR20** and **CHR21**, respectively, at a dose of 30 μM. This evidence suggests that each of these compounds could exert a neuroprotective effect and stimulated cell survival under low serum environment. However, further investigations are required to confirm the interpretation of this finding.

Although it is not obvious from data shown in Figure 3 that CHR21 is more potent than **CHR20**, we previously found that **CHR21** was more potent than **CHR20**, and that **CHR21** had greater neuroprotective effects against SNP-induced impairments in PC12 cells [25]. Therefore, **CHR21** was used as the representative compound in some of the following experiments in the current study.

### 2.3. Effects of **CHR20/CHR21** after SNP-Induced Cytotoxicity at Different Duration

**Figure 4:**
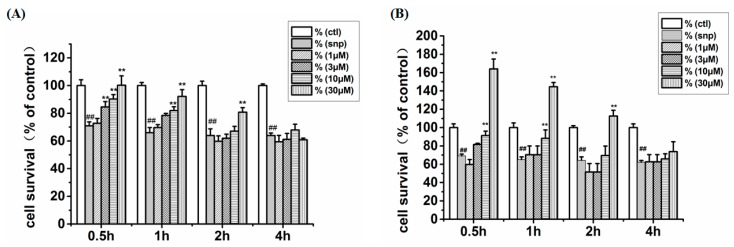
Effects of **CHR20/21** against SNP–induced insults in RGC-5 cells. RGC-5 cells were pre-treated with SNP at a concentration of 750 µmol/L for 0.5, 1, 2, and 4 h, respectively, then exposed to various concentrations of **CHR20** (**A**) and **CHR21** (**B**), respectively, for another 24 h. MTT activity was determined by MTT assay. The percentage of MTT activity was presented as mean ± SD for six replicates. ^##^ *p* < 0.01 *vs.* control group, ** *p* < 0.01 *vs.* SNP-treated group.

To investigate whether **CHR20/CHR21** can protect RGC-5 cells after SNP-induced oxidative damage at different durations, RGC-5 cells were pre-treated with SNP at a concentration of 750 µmol/L for 0.5, 1, 2, and 4 h, respectively. Afterwards, the cells were co-treated at various concentrations of **CHR20/CHR21** for another 24 h, respectively. Then the MTT activity was evaluated by the MTT assay. As shown in Figure 4, it was found that either **CHR20** or **CHR21** attenuated the SNP-induced injury in a dose-dependent manner after exposure to SNP for 0.5, 1, 2, and 4 h, respectively. For each dose, **CHR21** showed better neuroprotection than **CHR20**. The effect of **CHR21** was statistically significant at the concentration of 10 μmol/L (*p* < 0.05) and maximal neuroprotection was observed at 30 μmol/L. At this concentration, we also observed significant mitochondrial stimulation. When the duration of SNP exposure increased, the neuroprotective effects of **CHR20** and **CHR21** decreased. Milder effect was observed after exposed to SNP for 4 h.

It was interesting to find that the MTT activity of RGC-5 cells increased after a short duration of treatment with 750 µM SNP (0.5 and 1 h, respectively) followed by treatment with 30 μM of **CHR21**. We propose that short duration of 750 µM SNP following the treatment by 30 µM **CHR21** may have synergystic effects to stimulate the RGC-5 cells’ mitochondria activity. However, this observation requires further investigation. 

### 2.4. **CHR20** and **CHR21** Attenuated SNP-Induced Apoptosis of RGC-5 Cells

**Figure 5:**
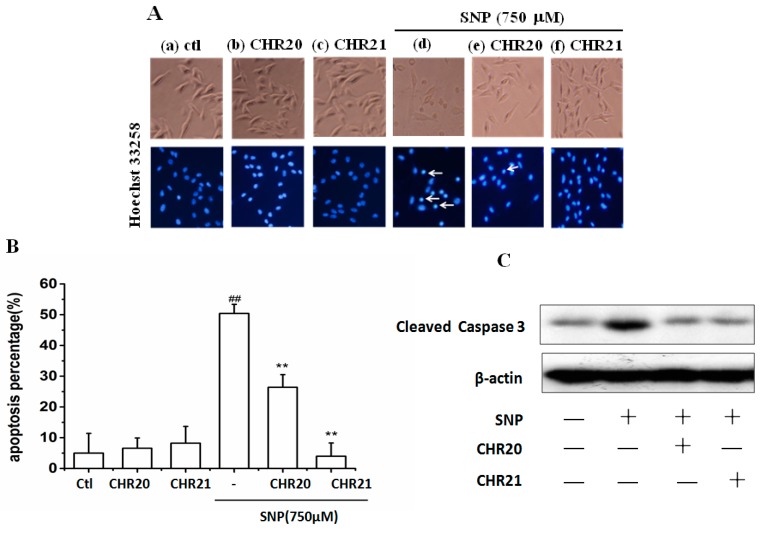
**CHR20** and **CHR21** protected RGC-5 cells from apoptosis induced by SNP. RGC-5 cells were pretreated with/without **CHR20/CHR21** for 2 h, respectively, after treatment of 750 μM SNP for 24 h and the cells were stained with Hoechst 33258 as described in Materials and Methods. (**A**) Morphological changes shown by fluorescence microscope (200×) image analysis. (a) control group; (b) 30 μM **CHR20** group; (c) 30 μM **CHR21** group; (d) 750 μM SNP group; (e) 750 μM SNP + 30 μM **CHR20** group; (f) 750 μM SNP + 30 μM **CHR21** group. The arrow indicates nuclear fragmentation or chromatin condensation; (**B**) Histogram showing the apoptosis rate in RGC-5 cells. The number of apoptic cells was about 1.5 × 10^4^ cells/well in 25 visual fields in 48-well culture plate, counted by high content screening system (ArrayScanVTI, Thermo Fisher Scientific, Waltham, MA, USA). The percentage of apoptotic cells was calculated as ratio of apoptotic cells and the total number of cells counted. Data are given as mean ± SD for three individual experiments. ^##^ *p* < 0.05 *vs.* control group. ** *p* < 0.05 *vs.* SNP group; (**C**) Effects of **CHR20/CHR21** on cleaved caspase-3. The expression of cleaved caspase-3 was determined by western blotting as described in Materials and Methods.

As shown in Figure 5A, cells in (a) to (c) groups looked normal. However, 750 µM SNP (d group) did induce apoptosis in RGC-5 cells. Interestingly, treatment with either **CHR21** or **CHR20** (30 μM), repectively, attenuated nuclear fragmentation, chromatin condensation, and cell apoptosis induced by SNP. **CHR20** decreased apoptosis rate from 50% ± 2.3% to 25% ± 3.2%; for **CHR21**, this rate was 4.5% ± 0.6% (Figure 5B).

To confirm that **CHR20/CHR21** did protect against apoptosis, caspase-3 activity was measured. Our result showed that SNP increased the amount of cleaved caspase-3 (active caspase-3), while pretreatment of **CHR20/CHR21** significantly attenuated this effect (Figure 5C).

### 2.5. The Effect of SNP and **CHR21** on the Activities of NOSs

To explore the role of NOS in the neuroprotective action of **CHR20** and **CHR21**, we examined the effect of SNP and **CHR21** on the activities of NOSs. To this end, RGC-5 cells were treated with SNP, with or without **CHR21**, and the activities of total NOS (tNOS), inducible NOS (iNOS) and constitutive NOS (cNOS includes nNOS and eNOS) were determined using a Typed Nitric Oxide Synthase (NOS) Detection Kit. Table 1 shows that SNP suppressed the activities of tNOS and cNOS while **CHR20** and **CHR21** significantly inhibited or blocked the effects of SNP. **CHR20/21** treatment, respectively, alone had no significant effect on iNOS but stimulated tNOS and cNOS activities. SNP significantly increased iNOS activity; while pre-incubation of RGC-5 cells with **CHR20/21** inhibited the effects of SNP on iNOS.

**Table 1:** The effects of SNP and **CHR21** on the activities of tNOS, iNOS, and cNOS ***^a^****.*

Groups	tNOS(U/mL)	iNOS(U/mL)	cNOS(U/mL)
ctrl	8.31 ± 0.44	4.63 ± 0.43	3.68 ± 0.12
CHR20	8.76 ± 0.50	4.61 ± 0.27	4.15 ± 0.17 **^&^**
CHR21	9.04 ± 0.60	4.54 ± 0.33	4.50 ± 0.18 **^&^**
SNP	7.54 ± 0.27 *	6.27 ± 0.13 *	1.27 ± 0.06 *
SNP + CHR20	9.78 ± 0.31 **^#^**	5.21 ± 0.24 **^#^**	4.57 ± 0.26 **^#^**
SNP + CHR21	9.86 ± 0.35 **^#^**	5.09 ± 0.26 **^#^**	4.77 ± 0.29 **^#^**

***^a^*** RGC-5 cells were treated with CHR21 (30 µM), SNP (750 µM), and CHR21 (30 µM) + SNP (750 µM), respectively. RGC-5 cells without addition of either CHR21 or SNP were set as the control group. The activities of tNOS (total NOS), cNOS (constructive NOS) and iNOS (inducible NOS) were tested by a Typed Nitric Oxide Synthase (NOS) Detection Kit. tNOS: total nitric oxide synthase; cNOS: constitutive nitric oxide synthase; iNOS: inducible nitric oxide synthase. U/mL: 1 nmol of NO formed/ml Cell Lysates in one minute. Activities of tNOS, iNOS and cNOS were tested by the Typed Nitric Oxide Synthase (NOS) Detection Kit. * *p* < 0.05 compared with ctrl group; **^#^** *p* < 0.05 compared with SNP group; **^&^** *p* < 0.05 compared with control group. (*n* = 3). All values are expressed as mean ± SD (*n* = 3).

### 2.6. **CHR20/CHR21** Increased the Phosphorylation Level of PI3K/Akt and MEK/ERK1/2 in RGC-5 Cells Time- and Dose-Dependently

**Figure 6:**
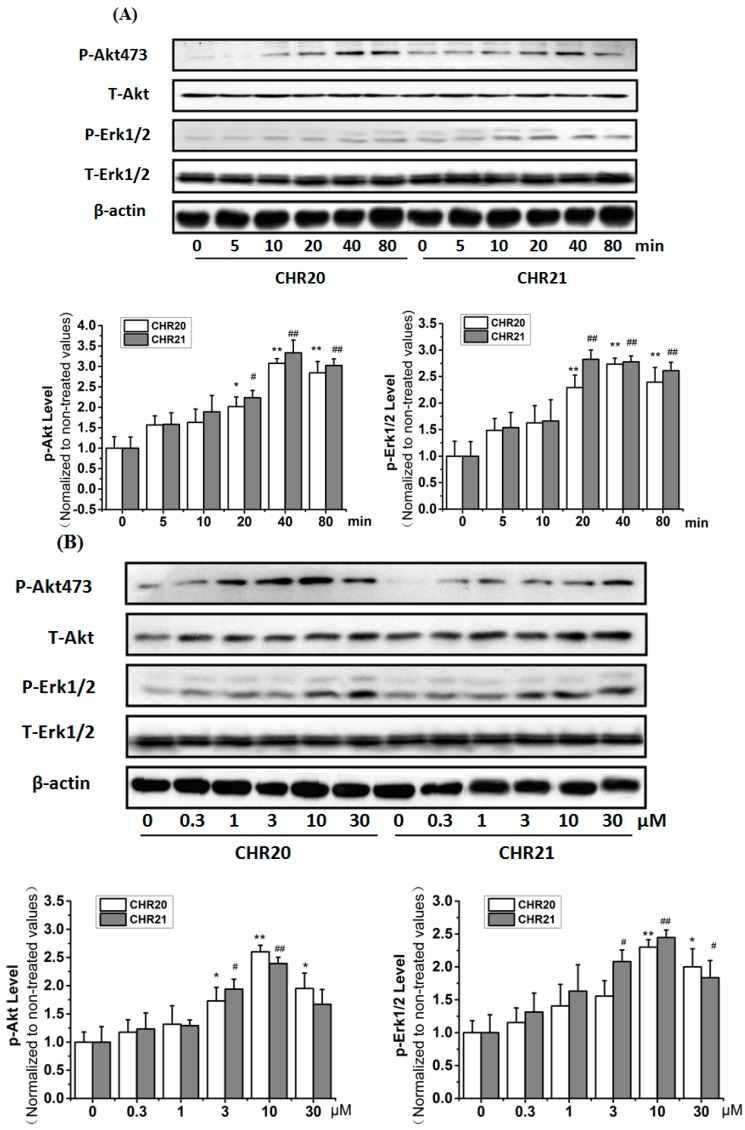

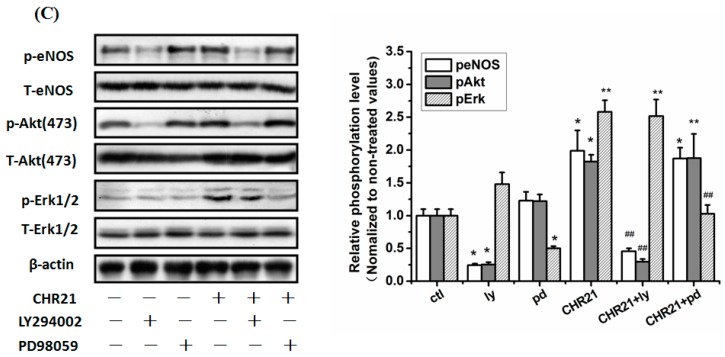
**CHR20/CHR21** increase phosphorylation levels in PI3K/Akt and MEK/ERK1/2. RGC-5 cells were treated with 10 μM **CHR20** and **CHR21**, repectively for 5–80 min or 0.3–30 μM **CHR20/CHR21** for 40 min. By applying Western blotting with antibodies including anti-phospho-Akt (Ser473), anti-phospho-ERK1/2, anti-phospho-and eNOS (Ser1177), respectively, the phosphorylation of the relevant proteins were determined. (**A**) **CHR20** and **CHR21** time-dependently increased the phosphorylation levels of Akt and ERK1/2 in RGC-5 cells; (**B**) **CHR20** and **CHR21** dose-dependently induced the phosphorylation of Akt and ERK1/2 in RGC-5 cells; (**C**) Effects of pathway inhibitors on the phosphorylation of Akt, eNOS and ERK1/2 affected by **CHR21**. The density of the blot in each lane was presented as mean ± standard deviation. Each data was calculated based on at least three individual experiments. * *p* < 0.05, ** *p* < 0.01 *vs.* control group. ^#^ *p* < 0.05, **^##^** *p* < 0.01 *vs.* **CHR21** pre-treated group. Blots were quantified using ImageJ software. pd: PD98059; ly: LY294002; p-eNOS: phosphorylated protein level of eNOS; pAkt: phosphorylated protein level of Akt; pErk: phosphorylated protein level of ERK; CHR: abbreviation of Chen HeRu; T-Erk: total protein level of ERK; p-NOS: phosphorylated protein level of NOS; T-NOS: total protein level of NOS.

As indicated in Figure 6A, the phosphorylation level of Protein kinase B (Akt) and extracellular signal-regulated kinase (ERK1/2) increased in a time-dependent manner after **CHR20**/**21** exposure. Increased phosphorylation level of these two kinases were found significant within 10–80 min, and peaked at 40 min. **CHR20**/**21** also increased the phosphorylation of Akt/ERK1/2 dose dependently (Figure 6B). The phosphorylation of Akt peaked at 10 µM for **CHR20**, and at 30 µM for **CHR21**. Quite interestingly, the phosphorylation of ERK1/2 peaked at 30 µM for **CHR20**, and at 10 µM for **CHR21**. Altogether it looks like that Akt is a little bit more sensitive to **CHR20**, while ERK1/2 is a little bit more sensitive to **CHR21**.

Pre-incubation with phosphatidylinositol 3-kinase (PI3K) inhibitor LY294002 (10 µM) completely abrogated **CHR21**-induced phosphorylations of Akt and eNOS, repectively; while pre-incubation with ERK1/2 inhibitor PD98059 (30 µM) for 30 min partially abrogated **CHR21**-induced phosphorylation of ERK1/2 (Figure 6C).

**Figure 7:**
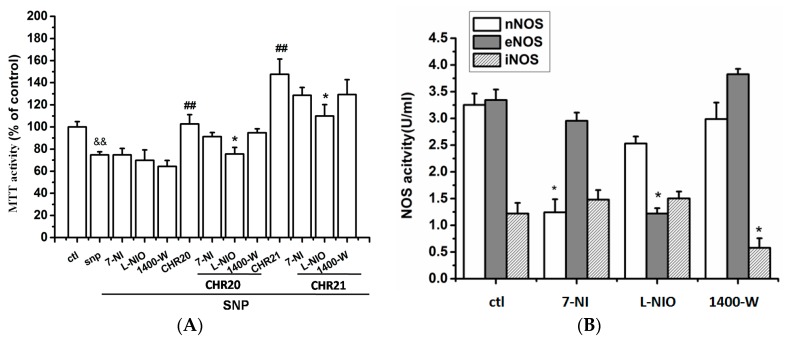
Effects of NOS on the action of **CHR21**. (**A**) Endothelial nitrioxide synthase (eNOS) mediates the action of **CHR21** on MTT activity in RGC-5 cells. Cells pre-exposed to 7-nitroindazole (7-NI, 50 μM, 30 min), an specific inhibitor for neuronal nitric oxide synthase (nNOS), *N*(5)-(-iminoethyl)-l-ornithine (l-NIO, 100 μM, 30 min), an inhibitor of endothelial NOS (eNOS), and compound 1400-W (20 μM, 30 min), a potent inhibitor of inducible NOS (iNOS), respectively, were treated with **CHR20/CHR21**. The MTT activity of cells determined by MTT assay. Only l-NIO attenuated the protective effects of **CHR20/21** on RGC-5 cells with statistically significant; while 7-NIO and 1400 W had no statistically significant effect. **^&&^** *p* < 0.01 *vs.* control; **^#^^#^** *p* < 0.01 *vs.* SNP group; * *p* < 0.05 *vs.* **CHR20** + SNP or **CHR21** + SNP (*n* = 3); (**B**) Effects of 7-NI, l-NIO and 1400 W on the NOS activities in RGC-5 cells. RGC-5 cells were treated with 7-NI, l-NIO and 1400 W, respectively. By applying Typed NOS Detection Kit, determination of the activities of nNOS, eNOS (endothelial NOS) and iNOS was carried out. One nmol of NO formed per mL cell lysate in one minute was defined as U/mL. With * *p* < 0.05 *vs.* control group, the difference is considered statistically significant. All values are expressed as mean ± SD (*n* = 3).

To determine whether the activation of NO synthase is involved, the role of various NO synthase subtypes in the protective effect of **CHR20**/**21** in RGC-5 cells were examined. RGC-5 cells were preincubated with various NOS subtype inhibitors following the treatment of **CHR20/21** with/without SNP, respectively. Afterwards, MTT activity was determined. As shown in Figure 7, it was shown that the inhibition of eNOS completely blocked the protective effect of **CHR20** (lane 8 to lane 4), however, only partialy to that of **CHR21** (lane 12 to lane 4). These results confirm the involvement of eNOS in **CHR20/21**-induced neuroprotection of RGC-5 cells. Furthermore, it seems that the inhibition of eNOS affected the neuroprotection of **CHR20** more than that of **CHR21**.

### 2.7. The PI3K/Akt/eNOS and ERK1/2 Signaling Pathways Are Involved in the Protective Effect of **CHR20/21** on SNP-Induced Apoptosis in RGC-5 Cells

**Figure 8:**
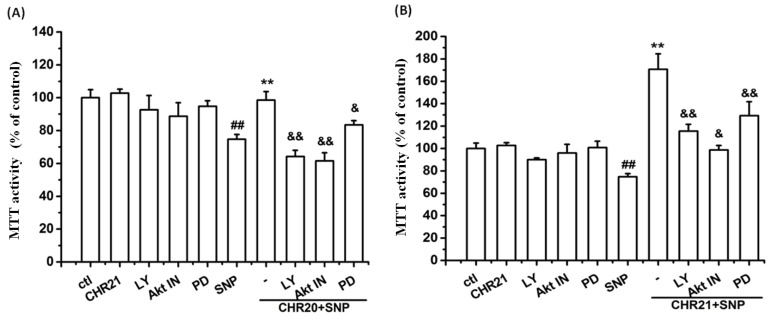

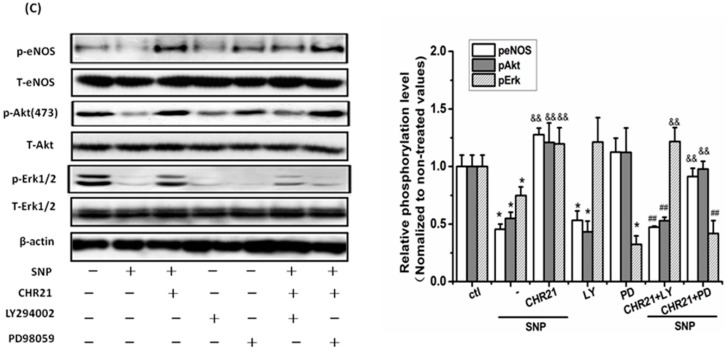
Both the PI3K/Akt/eNOS pathway and the ERK1/2 pathways were involved in the neuroprotective effects of **CHR20/21**. Pre-incubation of LY294002, Akt VIII inhibitor, and PD98059 at a dose of 10 µM, respectively blocked the neuroprotective effects of **CHR20** (**A**) and **CHR21** (**B**) in RGC-5 cells. MTT activity was determined by MTT assay. Each experiment was performed in triplicate. **^##^** *p* < 0.01 *vs.* SNP group; ** *p* < 0.01 *vs.* **CHR20/CHR21** + SNP group; ^&^ *p* < 0.05, ^&&^ *p* < 0.01 *vs.* **CHR20/CHR21** + PD group, or **CHR20/CHR21** + LY group, respectively; (**C**) SNP inhibited the phosphorylations of Akt, eNOS and ERK1/2, respectively. And LY294002 and PD98059 reversed the effects of **CHR21**. The density of each lane was presented as mean ± standard deviation for three individual experiments. * *p* < 0.05 *vs.* control group, **^&&^** *p* < 0.01 *vs.* SNP group, **^##^** *p* < 0.01 *vs.* **CHR21** + SNP group. Blots were quantified using Image J software. Similar results were obtained with **CHR20** (data not shown).

In order to explore whether the PI3K/Akt and ERK1/2 pathways were involved in the protective effect of **CHR20/CHR21**, LY294002 (10 µM), Akt VIII inhibitor (a specific Akt inhibitor) (10 µM), and PD98059 (10 µM) were applied. Cells were pretreated with them for 30 min, respectively, and then were treated with SNP (750 µM), following with or without treatment of **CHR20/21** for 24 h, respectively. As shown in Figure 8A, LY294002 and Akt VIII inhibitor (indicated as Akt IN) completely reversed the protective effects of **CHR20** with statistical significance; while PD98059 only partially reversed the protective effects of **CHR20**. Figure 8B indicated that LY294002, Akt VIII inhibitor, and PD98059 only partially reversed the protective effects of **CHR21**, respectively, in RGC-5 cells. It seemed that Akt VIII inhibitor was the more potent in reversing the protective effects of **CHR20**/**21**. Consistently, in Figure 8C, western blotting showed that SNP decreased the phosphorylations of Akt, eNOS, and ERK1/2, respectively; while **CHR21** reversed the inhibitory effect of SNP on the phosphorylations of Akt, eNOS, and ERK1/2, respectively. The effects of **CHR21** on the phosphorylation of Akt and eNOS were inhibited by the pre-incubation of LY294002 with RGC-5 cells; while the effect of **CHR21** on the phosphorylation of ERK1/2 was reversed by the pre-incubation of cells with PD98059.

### 2.8. Discussion

In the present study, we examined the effect of **CHR20/CHR21** on RGC-5 cell apoptosis induced by SNP, and also explored the cellular signaling mechanisms involved. We found that these two new genipin derivatives concentration-dependently protected RGC-5 from SNP-induced cell apoptosis. The protective effect of **CHR21** is significantly more potent than that of **CHR20**. Furthermore, we investigated the ability of **CHR20/21** to modulate different signaling pathways including ERK1/2 and PI3K/Akt/eNOS. Current results indicated that **CHR20/CHR21** time- and dose-dependently increased the phosphorylation of Akt (Ser473) and ERK1/2. SNP insult decreased the phosphorylations of Akt (Ser473) and ERK1/2 which was counteracted by **CHR21**. In another approach, the neuroprotective effects of **CHR20/CHR21** against apoptotic cell damage induced by SNP were abolished by a PI3K inhibitor, Akt inhibitor, eNOS inhibitor and ERK1/2 pathway inhibitor, together with a **CHR21**–induced increase in the phosphorylation of Akt (Ser473) and ERK1/2, respectively. Our results indicate that the activation of eNOS and the PI3K/Akt/ ERK1/2 pathways were involved in the neuronal protection of RGC-5 by **CHR20**/**21**.

Our previous results using MTT and Hoechst staining assays indicated that **CHR20**/**21** protected PC12 cells from apoptosis induced by SNP in a concentration-dependent manner [25]. In the present study, we showed that these two compounds exhibited the same effect to RGC-5 cells and were nontoxic to the cells even at the concentration as high as 30 µM. Both compounds showed the ability to increase the MTT activity of RGC-5 cells, which can be associated with increased cell viability under our experimental conditions [26,27]. This finding is consistent with the fact that they are tNOS and cNOS activators. They activate both enzymes to release NO endogenously, which stimulates cell growth without causing pathological effects. In fact, the roles of endogenous NO in cell survival and neuroprotection has been confirmed by many other studies. For example, when either l-arginine (the NO substrate) or 8-Bromo-cGMP (the cGMP analogue) was included in the culture medium, NGF-deprived neurons were rescued from apoptosis [29,30]. In the current experiment, when RGC-5 cells were cultured with SNP at a concentration ranging from 62.5 to 250 µM, MTT activity increased with SNP concentration, due to increased cell viability.

Pathological NO does increase the levels of ROS in many cell types and cause apoptosis. Interestingly, **CHR21/20** were capable of increasing mRNA levels of two anti-oxidative proteins, namely GCLC and SOD1, to antagonize the effects of ROS [26]. It has also been shown by other researchers that both ERK1/2 and PI3K are involved in the induction of antioxidant expression, implying that the activations of Akt and ERK1/2 are required for the activation of nuclear factor-like 2 (Nrf2) followed by up-regulating mRNA expression of antioxidant in rat vascular smooth muscle cells [31]. Because ERK1/2 and Akt are both protective factors distributed in the retina [32,33], it will be interesting to see whether ERK1/2 and Akt are involved in the neuroprotective effect of **CHR20/21** in RGC-5.**CHR20/21** in RGC-5 cells.

In the present studies, we clearly demonstrated that both PI3K/Akt and ERK1/2 signaling pathways were involved in the neuroprotective mechanism of **CHR20/21** to RGC-5 cells. High concentration (750 µM) of SNP inhibited PI3K/Akt and ERK1/2 in RGC-5 cells. Interestingly, **CHR20/21** reversed the inhibitory effect. They stimulated the phosphorylation levels of both Akt and ERK1/2. However, PD98059 (a specific MAPK pathway inhibitor), and LY294002 (a specific PI3K inhibitor), respectively blocked the phosphorylation of Akt and ERK1/2 stimulated by **CHR20/21** in RGC-5 cells, and attenuated the survival effects. Altogether, this evidence demonstrates that **CHR20/21** protects against SNP-induced impairments in RGC-5 cells via both signaling pathways of PI3K/Akt and ERK1/2. That SNP induces cell apoptosis is supported by the fact that the level of cleaved Caspase-3 is increased in the presence of 750 µM SNP. Interestingly, **CHR20/21** can protect against this apoptosis.

It was reported that SNP-induced vascular smooth muscle cells apoptosis was caused by cyclic guanosine monophosphate (cGMP)- and PI3K-involved inhibition of Bcl-2 down-regulation and/or cytochrome C-released caspase-3 activation cascades [34]. Therefore, as the activator of the PI3K pathway, **CHR20/21** certainly rescued cell apoptosis from pathological NO impairment.

Of course, both PI3K/Akt and ERK1/2 signaling pathways play important roles in the survival effect of many growth factors [35,36,37]. However, it is not necessary to stimulate ERK1/2 and PI3K simutaneously for cell survival. For example, although tropomyosin receptor kinase B (TrkB) activation *in vivo* may stimulate both ERK1/2 and PI3K pathways, it was confirmed that only the ERK1/2 pathway was involved to mediate the survival of axotomized RGCs [38]. Nevertheless, we believe that, as the dual activators of ERK1/2 and PI3K, **CHR20/21** might have more comprehensive neuroprotective effects to many neuronal insults.

No doubt, eNOS takes part in the neuroprotection of **CHR20/21**. As we know, eNOS releases endogenous NO, which is involved in the regulation of cell survival [39]. This enzyme is often regarded strictly as Ca^2+^/calmodulin-dependent. However, more and more evidence indicates that eNOS in the endothelium may be activated by stimuli such as IGF-1 [40], estrogen [41], shear stress [42], and isometric contraction [43]. This activation is independent of Ca^2+^ but phosphorylation-dependent by Akt [44]. It is likely that **CHR20/21** stimulate eNOS via the activation of Akt.

## 3. Experimental Section

### 3.1. Materials and Reagents

**CHR20/21** (Figure 1) were synthesized as described previously [26]; From Santa Cluz (CA, USA), 7-Nitroindazole, 1400-W and l-NIO were purchased; From the Centre of Cells Resource, Shanghai Institute of Life Science (Chinese Academy of Sciences, Shanghai, China), RGC-5 cells were obtained; From Sigma (Shanghai, China), poly-d-lysine, 3-(4,5-Dimethylthiazol-2-yl)-2,5-diphenyl tetrazolium bromide (MTT) and dimethylsulfoxide (DMSO) were purchased; From Gibco-BRL (Grand Island, NY, USA), fetal bovine serum (FBS) and RPMI-1640 medium were obtained; MDA detection kit, Sodium nitroprusside (SNP), BCA protein assay kit and Hoechst 33342 were purchased from Beyotime Institute of Biotechnology (Haimen, China); From the Cell Signaling Technology (Woburn, MA, USA), anti-phospho-Akt (Ser473), phospho-ERK1/2, anti-Akt, anti-ERK1/2 and anti-eNOS antibodies were collected; From Signalway Antibody (Pearland, TX, USA), anti-phospho-eNOS (Ser1177) antibody was purchased; From Calbiochem (Temecula, CA, USA), LY294002 (PI3K), Akt inhibitor, and PD98059 were obtained.

### 3.2. MTT Assay

MTT assay was performed according to protocols routinely used [45]. Briefly, after 24 h treatments, 11 μL MTT (5 mg/mL) and 100 μL of medium were added to each well after the aforementioned treatments. The plates were incubated for 3 h. After discarding the supernatants, 100 µL DMSO were added to each wells and mixed thoroughly. Incubation of the plates at 37 °C for another 10 min was made. Each sample was mixed once again, then the detection of the resulted formazan was made at 570 nm using a BIO-RAD680 plate reader (Thermo, Waltham, MA, USA). At least 3 parallel experiments were made and compared with the control.

### 3.3. Determination of NOS Activity

RGC-5 cells were cultured in RPMI-1640 medium containing 10% fetal bovine serum (FBS) in 5% CO_2_ and a humidified atmosphere of 95% at 37 °C for 24 h. Then the cells were treated with **CHR20**, **CHR21**, SNP, SNP + **CHR20** and SNP + **CHR21**, respectively for 24 h. The cells without the treatment of drugs were set as control for each time schedule. Following the removal of the medium, washing of the adherent cells with PBS for 1–2 times was carried out. Trypsin digestion of the cells was made and the cells were then passaged into an electro-polished seamless (EP) tube. Cells were then washed with fresh PBS. In order to remove trypsin, using low-speed centrifugation, the supernatants were discarded. To each EP tubes, 300 μL PBS were added. At an interval of 30 s to 1 min, ultrasonic radiation (power: 300 W; ultrasonic time: 3–5 s) disruption of the cells was made 4 times. During the whole ultrasonic process, the temperature was maintained within 0–5 °C by ice-water bath. Then activities of total NOS (tNOS) and constructive NOS (cNOS) were tested by the Typed Nitric Oxide Synthase (NOS) Detection Kit according to the manufacture’s instructions. The kit was purchased from Institute of Nanjing Jiancheng Bioengineering, Nanjing, China.

### 3.4. Detection of Apoptosis

RGC-5 cells were pre-treated with **CHR20** or **CHR21**, respectively, for 2 h before 750 μM/L SNP was added. Following washing with PBS, treatment of the cells with Hoechst 33342 (5 μg/mL) for 10 min at 37 °C were carried in the purpose of staining the cell. After that, removal of Hoechst 33342 was achieved by washing with PBS. By applying High content screening system (ArrayScanVTI, Thermo Fisher Scientific, Waltham, MA, USA), the apoptosis of cells were detected.

### 3.5. Cell Culture and Specific Signal Pathway Inhibition Treatment

RGC-5 cells were cultured at 37 °C in RPMI-1640 medium containing 10% fetal bovine serum (FBS) in the atmosphere of 5% CO_2_ and 95% humidity. By using trypsinization, the cells were passaged every 2–3 days. Culture media were replaced twice a week with fresh media. Stock culture was routinely subcultured at 1:5 ratio at a weekly interval. Solutions of **CHR20** and **CHR21** with various concentrations (3, 10, and 30 μmol/L) were added to the culture media for 2 h, following the addition of SNP (750 μmol/L) for 24 h. In the case of specific signal pathway inhibition experiments, RGC-5 cells were pretreated with LY294002, PD98059, and Akt VIII for 30 min, respectively, followed by the treatment of **CHR20** and **CHR21**, respectively before exposure to 750 μmol/L SNP for the next 24 h.

### 3.6. Western Blotting Assay

As the protocols described before [46], performance of Western blotting was carried on. According to the manufacturer’s instructions, proteins concentrations were determined by appling a BCA protein assay kit. On 8% polyacrylamide gels, samples containing equal amounts of proteins were analyzed, then were transferred to PVDF membrane, and probed with antibodies against antibodies including phospho-eNOS (Ser1177), phospho-Akt (Ser473), phospho-ERK1/2, Akt, ERK1/2,eNOS and caspase-3.

### 3.7. Statistical Analysis

All statistical analysis was performed based on the SPSS 16.0 statistical package. Data were expressed in the form of mean ± SEM. Statistical analysis for multiple comparisons was performed by analysis of variance (one-way ANOVA) with Kruskal–Wallis test; *p*-values <0.05 were considered statistically significant.

## 4. Conclusions

In summary, our data presented here show that **CHR20/21** increase MTT activity of RGC-5 cells at a concentration of 30 µM and protect against pathological NO-induced cell apoptosis by the activations of tNOS and cNOS, PI3K/Akt/eNOS and ERK1/2 pathways in an RGC-5 cell model. **CHR21** shows a stronger effect than **CHR20**. It was also found that **CHR20/21** increase the phosphorylation levels of PI3K/Akt and ERK1/2. Simutaneously, either specific Akt inhibitor or ERK1/2 inhibitor abolishes the stimulatory effects of **CHR20/21**, which supports the involvement of these kinases. Thus, **CHR20/21** may be a promising tool for further investigation of NO-induced neurodegenerative diseases.
